# COVID-19 Vaccines: An Overview of Different Platforms

**DOI:** 10.3390/bioengineering9020072

**Published:** 2022-02-12

**Authors:** Dmitry Kudlay, Andrey Svistunov

**Affiliations:** 1 Department of Pharmacology, Institute of Pharmacy, I.M. Sechenov First Moscow State Medical University (Sechenov University), St. Trubetskaya, 8, Building 2, 119991 Moscow, Russia; 2 I.M. Sechenov First Moscow State Medical University (Sechenov University), St. Trubetskaya, 8, Building 2, 119991 Moscow, Russia; svistunov_a_a@staff.sechenov.ru

**Keywords:** vaccine, prevention, vector, mRNA, peptides, COVID-19, boost

## Abstract

Vaccination is one of the key strategies to stop the COVID-19 pandemic. This review aims to evaluate the current state of vaccine development and to determine the issues that merit additional research. We conducted a literature review of the development of COVID-19 vaccines, their effectiveness, and their use in special patient groups. To date, 140 vaccines are in clinical development. Vector, RNA, subunit, and inactivated vaccines, as well as DNA vaccines, have been approved for human use. Vector vaccines have been well studied prior to the COVID-19 pandemic; however, their long-term efficacy and approaches to scaling up their production remain questionable. The main challenge for RNA vaccines is to improve their stability during production, storage, and transportation. For inactivated vaccines, the key issue is to improve their immunogenicity and effectiveness. To date, it has been shown that the immunogenicity of COVID-19 vaccines directly correlates with their clinical efficacy. In view of the constant mutation, the emerging new SARS-CoV-2 variants have been shown to be able to partially escape post-vaccination immune response; however, most vaccines remain sufficiently effective regardless of the variant of the virus. One of the promising strategies to improve the effectiveness of vaccination, which is being studied, is the use of different platforms within a single vaccination course. Despite significant progress in the development and study of COVID-19 vaccines, there are many issues that require further research.

## 1. Introduction

According to the World Health Organization (WHO), globally, as of 19 November 2021, there have been 255,324,963 confirmed cases of coronavirus disease 2019 (COVID-19), including 5,127,696 deaths [1]. Experts have observed a decline in economic growth of 3.4–7.6% in 2020; the rate is expected to return to pre-pandemic levels by 2024 at the earliest [2].

Epidemic control strategies, including public transport closures, switching to remote work, and full lockdowns turned out to be effective measures to combat the spread of the disease [3,4,5]. However, such measures greatly affect the global economy, therefore, they cannot be implemented for a long time.

During the pandemic, various COVID-19 treatment strategies have been developed and widely introduced to clinical practice. Antimalarials and some antivirals, such as lopinavir and ritonavir, which had been used at the early stages of the pandemic, were later found to be ineffective and/or associated with risks to patient health [6]. To date, an effective curative therapy that can be used in a wider population is still lacking. Therefore, only symptomatic and pathogenesis-based treatments are available, even for patients with moderate to severe disease [6,7,8,9]. In such a situation, the most promising strategy for overcoming the pandemic is vaccination of the entire population.

Data from the COVID-19 Vaccine Market Dashboard [10] shows that the average cost per COVID-19 vaccine dose ranges between USD 2 and 40 (Vaxzevria, AstraZeneca—USD 4.6, Ad26.COV2.S, Janssen—USD 9.6, Sputnik V, Gamaleya Research Institute—USD 17.38, BNT162b2, Pfizer/Biontech—USD 13.4, Spikevax, Moderna—USD 29.63, BBIBP-CorV, Sinopharm—USD 19.79, NVX-CoV2373, Novavax—USD 20.9). This represents a significant financial burden for low-income countries. A slower and delayed vaccination rollout in low- and middle-income countries has left them vulnerable to COVID-19 variants, new surges of the virus, and a slower recovery out of the crisis. High-income countries started vaccination, on average, two months earlier than low-income countries, and vaccination coverage in low-income countries is still strikingly low [11]. About 60.8% of the global population received at least one dose of a vaccine, while the vaccination rate in low-income countries was only 9.8% [12]. In European countries, 74.3% of the adult population have been fully vaccinated; in the meantime, in Russia, this rate has only been 47.6% [1]. Thus, as of the middle of January 2022, about 1800 versus over 4700 deaths per week have been reported in the UK and in Russia, respectively [1]. Over time, it becomes more important to consider vaccination of children and adolescents, as well as the most effective ways to use vaccines and to maintain their effectiveness to keep up with new mutations of the virus. At the same time, safety and efficacy of vaccines for COVID-19 prevention, as well as the strategies for using them in the general population and in special populations, are being extensively discussed. To identify research priorities, this review discusses the existing vaccine platforms and the main available data on the clinical use of vaccines.

## 2. Vaccine Platforms

**Figure 1:**
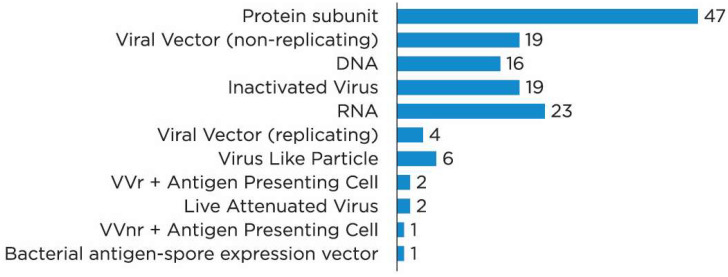
Platforms for vaccine products in clinical development [13]. Notes: DNA, deoxyribonucleic acid; RNA, ribonucleic acid; VVr, replicating viral vector; VVnr, non-replicating viral vector.

To date, 334 COVID-19 vaccines have been approved or are being developed worldwide; of these, 194 are in preclinical development and 140 are being investigated in clinical trials. This review discusses only the vaccines in clinical development. Figure 1. shows the platforms for vaccine development [13]. 

**Figure 2:**
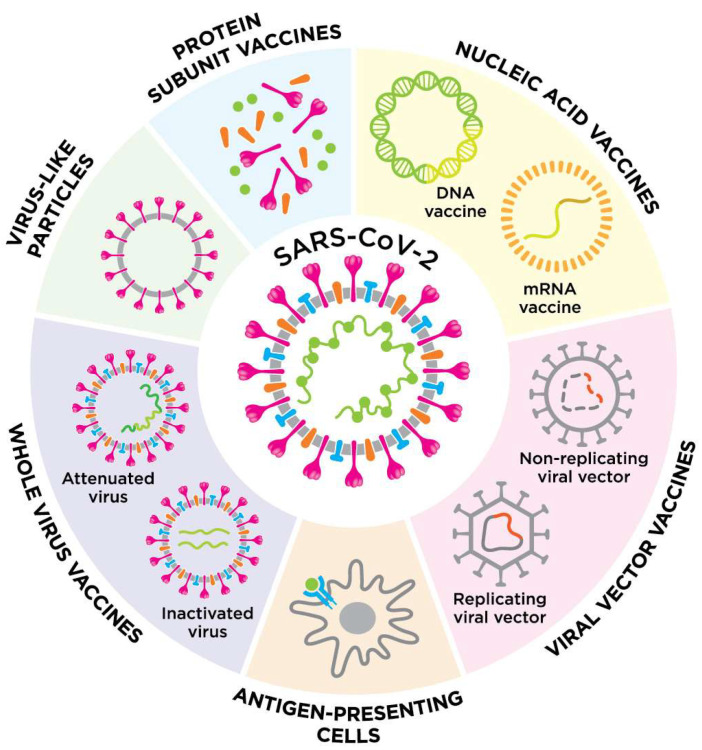
An overview of the different vaccine platforms against COVID-19.

The majority of the vaccines are protein subunit (PS) vaccines, also called peptide vaccines. The ribonucleic acid (RNA) vaccines, as well as inactivated virus (IV), non-replicating viral vector (VVnr), and deoxyribonucleic acid (DNA) vaccines are also common (Figure 2). There are also single vaccines containing a viral vector that can replicate (replicating viral vector, VVr) virus-like particles (VLP) VVr with antigen-presenting cells (APC), live attenuated virus, VVnr with APC, or bacterial antigen–spore expression vector, in clinical development. In the meantime, 33 vaccines have been approved in at least one country (Table 1) [14]. 

## 3. Vaccines Containing Non-Replicating Viral Vector

VVnr vaccines contain SARS-CoV-2 genetic material encapsulated in a viral (vector) envelope; the vector has the ability to enter human cells, but it cannot replicate there. Viral vectors have been produced and used in medicine for a long time. Initially, vectors were more popular in gene therapy; however, they have been used to develop vaccines for more than 25 years [15]. The most common vectors include adenoviruses, herpes viruses, alphaviruses, as well as measles, variola, and vesicular stomatitis viruses. Classical vectors (measles and variola viruses) have some obvious advantages, including persistent or even lifelong immunity; however, their use is currently limited by the difficulties in controlling the production of these vectors and the relatively high risk of virus mutations during the manufacturing [16]. 

At the time of this review, all registered VVnr vaccines are based on one or more of the following adenoviral vectors:

- Vaxzevria/Covishield vaccine is composed of ChAdOx1 chimpanzee adenovirus vector (previously known as ChAdY25) [17,18].
- Ad26.COV2.S vaccine contains recombinant human adenovirus type 26 (Ad26) vector [19,20].
- Sputnik V and Sputnik Light vaccines are based on recombinant adenovirus type 26 and type 5 (Ad26 and Ad5) vectors [21,22,23].
- Ad5-nCoV vaccine contains Ad5 adenovirus vector [24].

All vaccines listed above are two dose (two vaccine doses are required for complete vaccination), except for the Janssen and Sputnik Light vaccines, which are administered once. All vaccine vectors carry a viral RNA fragment that encodes a viral spike (S) protein, which mediates the binding and translocation of the virus into the cell. Thus, the synthesis of the SARS-CoV-2 antigen occurs in human cells after the administration of a vaccine [25].

Vector vaccines have a number of clinical advantages. VVnr vaccines elicit a relatively persistent and potent immune response [16], thus providing long-term protection against infection and severe disease. Even a single VVnr vaccine injection is associated with sufficient immunogenicity to achieve protective antibody titers [26]. This allows for a development and approval of Sputnik Light and Ad26.COV2.S single-phase vaccines. A hypothesized decrease in the immune response in the presence of natural immunity to the vector or vector-like viruses has not been confirmed in the studies [27]. In the meantime, the problem of preserving VVnr immunogenicity upon repeated administration of vaccines remains relevant [28]. The first way to solve the problem is the alternation or use of different vectors, which has been implemented in the Sputnik V vaccine [21]. The second way to avoid vector neutralization is to use well-known technologies, including pegylated forms [29], which are currently in preclinical development and are mainly used in gene therapy [30,31], or dosage forms with microspheres [32]. Another significant drawback faced by many companies is the difficulty in scaling up the production of vector vaccines due to the peculiarities of vector production that require replication in cells cultured on solid substrates. Moreover, the direct process of assembling the vector with SARS-CoV-2 genetic material limits the scalability of production. 

Despite the existing difficulties in production and not fully resolved problems of long-term efficacy and its maintenance upon repeated administrations of the same vaccine, VVnr is one of the most promising platforms for the development of new COVID-19 vaccines and the modification of existing ones. 

## 4. Vaccines Containing Viral Ribonucleic Acid

The COVID-19 pandemic has fast tracked the development of new ribonucleic acid (RNA) vaccine technology. Vaccines based on nucleic acids act due to the delivery of genetic material encoding immunogenic fragments of the virus into human cells. After the delivery of genetic material (RNA or deoxyribonucleic acid (DNA), viral proteins are synthesized, initiating the immune response and the synthesis of antibodies to the virus [33]. There are three main technologies for the development of nucleic acid vaccines:

- Vaccines that contain DNA (DNA vaccines). When reaching the host cells, DNA is transcribed into messenger RNA, followed by protein synthesis [33].
- Vaccines that contain messenger RNA (mRNA). The use of mRNA helps bypass transcription and simplify the synthesis of antigens [33].
- Vaccines based on mRNA, capable of self-amplification due to the additional inclusion of nsP1-4 sequence of alphavirus protein in the original RNA, which amplifies the coding region of the antigen mRNA; another option is the use of trans-amplifying mRNA, where nsP1-4 mRNA is a separate fragment [34].

DNA vaccines are considered a less promising platform due to the presence of additional stages in the immune response formation, which may be excluded with RNA vaccines. However, the first DNA vaccine for COVID-19 (ZyCoV-D) has been approved in India [35]. 

Self-amplifying RNA elicits a more potent immune response and requires lower doses [36]; however, simpler methods of immunization have apparently been preferred due to the necessity of a rapid development of COVID-19 vaccines. Pfizer and Moderna vaccines contain an mRNA fragment that encodes an entire S protein containing the largest number of immunogenic epitopes of the virus [37,38]. The development and production of RNA vaccines are associated with two significant technological barriers. RNA vaccines require the use of delivery systems that can protect genetic material from the host organism until it is delivered to target cells. In the two approved RNA vaccines (BNT162b2 and mRNA-1273), the problem has been solved by lipid nanoparticles for RNA delivery [37,38]. Another problem is easy RNA degradation in the external environment [33]. To stabilize mRNA and prevent its degradation, modified nucleosides have been incorporated into the existing vaccines [37,38]. However, the use of modified nucleosides does not completely solve the problem of mRNA instability and easy degradation. Therefore, mRNA vaccines require supply chains with strict requirements for ultra-low temperatures for transportation and storage [39]. 

Overall, mRNA is a promising and practically effective technological platform. At the same time, the main challenge for mRNA vaccines remains the stability of mRNA during production, transportation, and administration to patients. To increase the availability of mRNA vaccines, this problem has to be overcome. 

## 5. Vaccines Containing Viral Proteins

Virus proteins with immunogenic potential as an active component of vaccines are a well-developed technological platform; using this platform, a number of vaccines have been developed for the prevention of infectious diseases, including hepatitis B and C, influenza, pertussis, and human papillomavirus [40]. According to WHO, 47 and 75 vaccines containing SARS-CoV-2 proteins are in clinical and preclinical development, respectively [13]. 

To develop COVID-19 vaccines, the entire S protein (Nuvaxovid (NVX-CoV2373), MVC-COV1901) or its receptor-binding domain (RBD) (Abdala (CIGB-66)), which is responsible for the binding of the virus to the angiotensin-converting enzyme (ACE) 2 receptor, are mainly used [41]. The WHO considers the EpiVacCorona vaccine to be a protein vaccine, but it contains peptide fragments of the SARS-CoV-2 S protein [42]. The results of studies on the effectiveness of the EpiVacCorona vaccine have not yet been published; only data from an independent study are available [43]. 

The advantages of protein vaccines include the streamlined production and more acceptable stability in the external environment [40]. On the other hand, in most cases, protein vaccines require an adjuvant to boost the immune response [44]. In addition, some experts suggest a possible Th-1/Th-2 response polarization with subunit vaccines [45]; however, the S protein induces a balanced immune response from CD4+ and CD8+ T-lymphocytes with maintained synthesis of antibodies, including virus-neutralizing ones [46].

The development of protein vaccines is associated with a long search for a sufficiently immunogenic target to be introduced into a vaccine. Despite a large number of candidate vaccines developed using this platform, only several vaccines have been registered to date, mainly in the countries of origin; the rest of the vaccines are to be registered in case of successful completion of phase 2/3 clinical trials. 

Overall, protein vaccines are a well-studied platform that has the necessary prerequisites for production and use. At the same time, the manufacturers face obstacles in vaccine approval that need to be overcome.

## 6. Vaccines Containing Inactivated Virus

Inactivated vaccines, as well as live attenuated vaccines, are the platforms with the longest history. To date, according to the WHO, 10 inactivated vaccines have been registered for COVID-19 prophylaxis, with two vaccines (BBIBP-CorV and CoronaVac) being registered in 88 and 53 countries, respectively, making them one of the most widely used vaccines. 

Due to the relative simplicity of the inactivated vaccine production and the possibility of rapid deployment of mass production and appropriate adjustment of the technological process (for example, changes in a virus strain), this technology is still widely used [47,48]. At the same time, the production of inactivated vaccines requires thorough batch control, since an impairment of inactivation can lead to a massive wave of infection [48]. Another significant disadvantage of inactivated vaccines may be the relatively low immunogenicity compared to that of mRNA or vector vaccines. One study showed that neutralizing antibody titers after vaccination with the Pfizer vaccine were significantly higher than in patients who received the CoronaVac vaccine [49]. On the one hand, a decrease in immunogenicity may lead to lower clinical efficacy, but on the other hand, these vaccines are more appropriate for medically compromised patients, since the risk of adverse reaction is a more significant factor for choosing a vaccine for such patients compared to the general population.

Inactivated vaccines are a well-established platform; however, the manufacturing process requires thorough control of virus inactivation, and further modifications of approved or developed vaccines may be required to improve their immunogenicity and efficacy. 

## 7. Other Promising Platforms 

Among other technological platforms used for vaccines in clinical development, antigen-presenting cell (APC) vaccines are of particular interest. APC-based vaccines contain laboratory dendritic cells, which carry the viral antigen on their surface. Thus, after the administration of the vaccine to the patient, the process of antigen presentation to the host’s immune cells and the formation of post-vaccination immunity are significantly accelerated [50]. To date, the technology is mainly being developed in oncology for the treatment of solid tumors. APC vaccines for COVID-19 prophylaxis are being studied in clinical trials [51]. 

## 8. Delivery of Different Types of Vaccines

According to the guidelines of the World Health Organization (WHO) [13], most COVID-19 vaccines are designed to be delivered by the injectable route. However, the oral route of administration (Vaxart’s Oral Mucosal COVID-19 vaccine, IosBio’s OraPro-COVID-19™ vaccine) has been described. The oral tablet versions of the COVID-19 vaccine would be used to reach regions where proper healthcare staff or professionals are not available, especially in the underdeveloped countries. The tablet form of the vaccine would aid a common person with no access to a proper vaccination site, as it would eliminate the need for any healthcare professional, as well as the fear of common side effects of the injectables, such as malaise, pain, and inflammation [52]. 

## 9. Immunogenicity and Safety of Vaccines

The immunogenicity of a vaccine is a key parameter reflecting its effectiveness. According to the WHO and the European Medicines Agency (EMA) guidelines, immunogenicity must be assessed in clinical development programs of all vaccines before initiating effectiveness studies. The guidelines recommend the assessment of not only the effective immunogenicity (titers of neutralizing antibodies), but also the effect of vaccines on T-cell immunity to evaluate potential long-term efficacy [53,54].

The Pfizer and Sinovac vaccines may be an example of the relationship between the immunogenicity of COVID-19 vaccines and their effectiveness. The administration of the mRNA vaccine led to the formation of significantly higher titers of virus neutralizing antibodies [49], which were associated with effectiveness in preventing COVID-19; the effectiveness of Pfizer and Sinovac vaccines is 95 and 83.5%, respectively [55,61]. An in silico study evaluated the effect of neutralizing antibodies synthesized following the administration of various vaccines on their clinical effectiveness. The most immunogenic vaccines were Pfizer, Moderna, and Novavax, which were associated with antibody titers 2–4 times higher than those obtained in convalescents, as well as the Sputnik vaccine, which led to antibody titers 1–1.5 times higher than those in convalescents. Following the administration of AstraZeneca or Janssen vaccines, the antibody titers were two times lower than those in the convalescents. As for the CoronaVac vaccine, the amount of antibodies was about six times lower than that following the infection. Mathematical modeling showed that the titers of neutralizing antibodies after vaccination linearly correlated with the effectiveness of the vaccines [62]. 

Immunogenicity can influence directly the safety and tolerability profile of vaccines. In clinical trials, the incidence of adverse events was significantly higher following the administration of mRNA and vector vaccines (local reactions in 40–89% of cases and systemic reactions in 44–86% of cases) than after the injection of inactivated vaccines (injection site reactions in 5–23% of cases, systemic reactions in 4–18% of cases) [63]. 

The immunogenicity of vaccines characterizes their clinical effectiveness (Table 2). At the same time, immunogenicity determines the safety and tolerability of vaccines, which is essential when choosing a vaccine for vulnerable populations.

## 10. Vaccine Selection Approaches

### 10.1. General Population

The US Centers for Disease Control and Prevention (CDC) consider COVID-19 vaccines registered in the US (Pfizer/Biontech, Moderna, Janssen) to be interchangeable and equivalent in the general population [64]. The WHO considers the AstraZeneca/Oxford, Johnson & Johnson, Moderna, Pfizer/BionTech, Sinopharm, Sinovac, COVAXIN, Covovax and Nuvaxovid vaccines to be safe and effective. These vaccines are equivalent in clinical practice, and healthcare professionals should use any available option [65]. The Australian guidelines prefer Moderna or Pfizer/Biontec vaccines in patients under 60 years over AstraZeneca [66]. 

### 10.2. Pregnant Women

In the US, vaccination of pregnant women is approved for all registered vaccines. According to the CDC, the most data on the efficacy and safety of COVID-19 vaccines in pregnant women is available for the Pfizer/Biontech vaccine [64]. In Australia, mRNA vaccines are recommended for pregnant women and nursing mothers [66]. The Russian national guidelines agree with the foreign regulators; however, they emphasize that the data on the safety of vaccines in nursing women are currently not enough for an unambiguous judgment about the safety of vaccines in this population [67]. 

### 10.3. Children and Adolescents

The US Food and Drug Administration (FDA) recommends the Pfizer/Biontech vaccine for children aged 12 to 17 years [68]. The WHO shares this opinion [65]. In Russia, COVID-19 vaccines in children and adolescents are being evaluated [67]. 

### 10.4. Patients with a History of COVID-19

The CDC recommends vaccination of patients with active COVID-19 infection after complete clinical recovery [64]. In a recent study, it was found that in patients who had had COVID-19, the levels of total and virus neutralizing antibodies were significantly higher than in patients who had had no contact with SARS-CoV-2 antigens [69]. Similar results were obtained for the AstraZeneca vaccine [70]. 

### 10.5. Patients with Compromised Immune System

The CDC recommends mRNA vaccines or the Janssen single-dose vaccine for immunocompromised patients. For mRNA vaccines, an additional vaccine dose is recommended for patients with immune suppression [64]. The Pfizer vaccine was approximately 20% less effective in patients with immune suppression compared to the general population [71]. The Canadian guidelines recommend that patients with primary immunodeficiency be vaccinated with mRNA vaccines, given the risk of more severe infection [71,72]. Vaccination is also approved for patients with primary immunodeficiency and a history of COVID-19 [73]. 

Available data suggest that widely available COVID-19 vaccines are generally interchangeable. Messenger RNA vaccines are considered to be safe in children and pregnant women. However, due to the short-term clinical use and the lack of convincing results, the following questions require further investigation:

- Which vaccines are safer for patients over 60 years and those with severe concomitant diseases?
- How will the modification of vaccines for new coronavirus strains affect their clinical use?
- Is it possible to extrapolate the results of safety assessment of the Pfizer vaccine to the Moderna vaccine?

## 11. Vaccination and SARS-CoV-2 Mutation

Several authors pointed out the ability of SARS-CoV-2 to acquire mutations that can significantly reduce the effectiveness of vaccination [74,75]. To date, there have been six main variants of the virus that differ from the original virus found in China. The Alpha variant was first discovered in the UK in September 2020. This variant of SARS-CoV-2 includes viral strains characterized by 23 mutations, including 7 mutations and 2 deletions in RNA fragments encoding the S protein [76]. This variant is characterized by significantly increased virulence and an increased risk of death or severe course of COVID-19 [77]. The Beta variant was first identified in the Republic of South Africa in December 2020. Mutations in strains belonging to the Beta variant include 9 mutations and 1 deletion in S protein RNA [76]. The variant is characterized by a significant acceleration of spread of the disease compared with the reference strain [78]. Another variant of SARS-CoV-2, called Gamma, was found in patients arriving from Brazil in early 2021. In this variant, 12 mutations modify the structure of the S protein, leading to an increase in virulence [76]. For the Delta variant, discovered in India in October 2020, there are 19 known S protein mutations, with 2 main mutations, L452R and E484Q, altering the structure of the RBD domain of the S protein. This variant is also characterized by an accelerated spread of the infection and the risk of more severe disease [76]. On 26 November 2021, the WHO designated variant B.1.1.529, called Omicron, as a Variant of Concern (VOC). This decision was based on evidence provided by the WHO Technical Advisory Group on SARS-CoV-2 Virus Evolution (TAG-VE) that Omicron had several mutations that could affect its characteristics, such as how easily it spreads or the severity of the disease it causes [79]. The main concerns with Omicron are whether it is more infectious or serious than other VOCs and whether it might bypass vaccine-induced immunity. 

Mutations in the S protein and, in particular, the RBD domain, are associated with a significant decrease in the neutralizing activity of antibodies in convalescents or vaccinated people (79). Clinical data indicated different effects of SARS-CoV-2 mutations on the efficacy of mRNA vaccines. A scientific review showed that some coronavirus variants may reduce the efficacy of neutralizing vaccine-induced antibodies, but the effect may be clinically non-significant [76]. 

A real-world study showed that the effectiveness of the Pfizer and AstraZeneca vaccines against the SARS-CoV-2 delta strain was reduced; however, the differences were clinically non-significant. Following the administration of a single dose of the vaccine, the effectiveness against the Alpha and Delta variants was 48.7% and 30.7%, respectively. The administration of two doses of the Pfizer vaccine led to 93.7% protection against the infection caused by the Alpha variant and 88% protection in case of the Delta variant infection. For the AstraZeneca vaccine, the respective values were 74.5 and 67% [80]. CoronaVac vaccine was also less effective against the Delta variant; in a Chilean study, it was 67% effective in case of symptomatic Alpha variant infection, while in another real-world study, it was 59% effective against the Delta strain [81]. In preclinical studies, Sputnik V vaccine showed high plasma activity against the Alpha variant and a moderate decrease in neutralizing activity against the Beta variant [82]. The efficacy of this vaccine in preventing hospitalization of patients with the Delta variant was 81% [83]. 

Subunit vaccines are potentially most susceptible to virus mutations, since they contain individual components of the virus or fragments of its proteins. For example, the effectiveness of the Novavax vaccine, which included the RBD domain, was two times lower in case of the Beta variant compared to the original SARS-CoV-2, being 50% [84]. At the same time, it should be noted that the WHO-recommended threshold for vaccine effectiveness was 50%, thus, despite the decrease in efficacy against the Delta strain, the level of protection still exceeded the recommended values to support the use of the vaccine. 

Most COVID-19 vaccines target the S protein, which contains the largest number of immunogenic epitopes. To date, the approved vaccines remain effective; however, significant changes in the structure of the S protein may lead to temporary absence of effective vaccines. One of the options for creating more universal vaccines that are effective over a long period of time is the creation of vaccines aimed at induction of an immune response to the internal SARS-CoV-2 antigens. This will help ensure a stable T-cell response and protect the vaccine against rapid mutation of the S protein [85]. 

## 12. Booster SARS-CoV-2 Immunization

### 12.1. Booster Immunization Approaches 

The CDC recommends a booster shot for patients ≥65 years of age, long-stay patients, and patients aged 50–64 with concomitant diseases. It is recommended to administer the booster dose 6 months after completing the primary vaccination [86]. According to recent data, the Pfizer mRNA vaccine may provide relatively persistent immunity, therefore, some experts oppose booster immunization 6–12 months after the primary vaccination, as recommended by the vaccine developer [87]. In the UK, booster immunization with mRNA vaccines is currently recommended 6 months after completing the primary vaccination [88]. The EMA also states that booster doses are not required for patients from the general population who have received a full course of one of the approved vaccines [89]. 

At the time of this review, one of the issues that was being exhaustively discussed by both scientists and clinicians was the ethical aspect of booster immunization. In low-income countries, there is currently a significant shortage of vaccines. Some clinicians point out the need to limit booster dosing to provide vaccines to the population of developing countries [90]. The world statistical data show that booster immunization is used in few countries, including the US, Russia, France, Germany, Turkey, and Chile, while the latter is the leader in the number of booster immunizations [91]. Notably, at the same time, the global rate of vaccination has decreased from 0.55 dose per 100 people/day at the end of June 2021 to 0.34 dose per 100 people/day at the end of January 2022 [92]. However, only 61% of the global population received at least one dose of a vaccine [93]. 

At the same time, the main regulatory authorities, as indicated above, do not recommend booster immunization for the general population. To determine the rationale and the need for additional doses of the vaccine, longer monitoring of vaccine efficacy in real-world clinical practice is necessary.

### 12.2. Combination and Sequential Administration of Vaccines

To date, data on the sequential administration and combination of different vaccines are very limited. One study found that the use of the AstraZeneca vaccine as the first dose and the Pfizer vaccine as the second one resulted in a significant increase in the immune response. In patients who received a combination of vaccines, an increased synthesis of antibodies to the RBD domain and S protein was noted. A 4-fold increase in the cellular response to the vaccine was also observed compared to that noted following the administration of a single AstraZeneca vaccine dose. At the same time, a slight increase in the frequency of injection site reactions was noted [94]. There were some reports on a study of the AstraZeneca and Sputnik V vaccine combination. However, the results of the study have not yet been published [95]. 

In Cambodia, the national regulatory authority recommended the use of Sinovac vaccine as a booster vaccine for patients who had previously received AstraZeneca. Denmark suggests a vaccination course including AstraZeneca VVnr vaccine and one of the mRNA vaccines [96]. Thus, the approaches to booster immunization and the views on its rationale may differ significantly between countries. As demonstrated by the above data, the regulatory authorities do not recommend the combined use of VVnr vaccines, which is most likely due to the risk of a decrease in the immunogenicity of these vaccines. As stated above, all VVnr vaccines registered in one or more countries are based on only three different adenoviral vectors.

To find out the real need for repeated use of COVID-19 vaccines, it is necessary to perform epidemiological monitoring in the vaccinated populations for a longer period of time, as well as to obtain the results of studies evaluating the effectiveness and safety of vaccine combinations.

## 13. Conclusions

To date, there have been three following platforms playing a crucial role in the global strategy for COVID-19 vaccination: mRNA, VVnr, and inactivated virus vaccines. All of these platforms have some technological advantages and disadvantages:

- VVnr is a well-developed platform; however, these vaccines are technologically difficult to produce, and their long-term effectiveness is considered questionable by some expert groups.
- Messenger RNA vaccines have proven to be a technological breakthrough during the COVID-19 pandemic, as this class of vaccines was first approved for the COVID-19 prophylaxis. They have shown good immunogenicity; however, they require special conditions for transportation and storage that lead to certain limitations on the conditions of their use.
- Inactivated vaccines are a classic platform. Despite the apparent simplicity of inactivated vaccine production, batch quality control remains a key issue for their use. The potential long-term efficacy of inactivated vaccines is also questionable.
- All common vaccines have been proven to have acceptable clinical efficacy, according to the WHO guidelines. However, it is becoming more important to consider vaccination in the following special patient groups: children and adolescents, pregnant women, the elderly, and patients with concomitant diseases, including those with impaired immunity. The use of vaccines in these groups of patients requires further research in order to provide guidelines for routine clinical practice. The effect of new SARS-CoV-2 mutations on the immunogenicity of vaccines and the long-term effectiveness of COVID-19 vaccination are also to be investigated, since it can only be established in long-term observational studies.

Overall, various groups of scientists and pharmaceutical companies have successfully met the challenge of developing effective and safe vaccines against COVID-19. We believe that the more vaccine platforms, the greater the potential for infection control. However, if necessary, the vaccines will need to be updated. However, there are still many issues that warrant further study. 

## Figures and Tables

**Table 1:** COVID-19 vaccines approved by ≥1 countries [14].

Platform	Vaccine Name	Manufacturer	No. of Countries, Where a Vaccine Was Approved
VVnr	Ad26.COV2.S	Janssen, Beerse, Belgium	106
	Sputnik V	Gamaleya National Center of Epidemiology and Microbiology, Moscow, Russian Federation	74
	Sputnik Light		24
	AZD1222, Vaxzevria	Oxford University/AstraZeneca, Södertälje, Sweden	137
	AZD1222, Covishield	Serum Institute of India, Pune, India (based on AstraZeneca technology)	47
	Ad5-nCoV, Convidecia	CanSino, Tianjin, People’s Republic of China	10
RNA	BNT162b2, Comirnaty	Pfizer/Biontech, Mainz, Germany	137
	mRNA-1273, Spikevax	Moderna, Cambridge, MA, USA	85
	TAK-919	Takeda, Tokyo, Japan (based on Moderna technology)	1
PS	CIGB-66, Abdala	Cuban Center for Genetic Engineering and Biotechnology, Havana, Republic of Cuba	6
	EpiVacCorona	“Vector”, National Research Center for Virology and Biotechnology, Novosibirsk, Russian Federation	4
	MVC-COV1901	Medigen, Taipei, Taiwan	2
	ZF2001	Anhui Zhifei Longcom, Beijing, People’s Republic of China	3
	Corbevax	Biological E Limited, Hyderabad, India	1
	Aurora-CoV (EpiVacCorona-N)	“Vector”, National Research Center for Virology and Biotechnology, Novosibirsk, Russian Federation	1
	Soberana 02	Instituto Finlay de Vacunas Cuba, Havana, Republic of Cuba	4
	Soberana Plus		1
	Recombinant SARS-CoV-2 Vaccine (CHO Cell, NVSI-06-08)	National Vaccine and Serum Institute, Beijing, People’s Republic of China	1
	Nuvaxovid (NVX-CoV2373)	Novavax, Gaithersburg, USA	32
	Razi Cov Pars	Razi Vaccine and Serum Research Institute, Karaj, Iran	1
	COVOVAX (Novavax formulation)	Serum Institute of India, Pune, India	3
	SpikoGen, COVAX-19	Vaxine/CinnaGen Co., Tehran, Iran	1
IV	Covaxin	Bharat Biotech, Hyderabad, India	13
	KoviVac	Chumakov Federal Scientific Center for Research and Development of Immune-and-Biological Products of Russian Academy of Sciences, Moscow, Russian Federation	3
	QazVac	Research Institute for Biological Safety Problems, Guardeyskiy, Republic of Kazakhstan	2
	KCONVAC (Vero Cells), KconecaVac	Minhai Biotechnology Co., Beijing, People’s Republic of China	2
	COVIran Barekat, COVID-19 Inactivated Vaccine	Shifa Pharmed Industrial Co, Karaj, Iran	1
	Covilo, BBIBP-CorV (Vero Cells)	Sinopharm (Beijing), People’s Republic of China	88
	Inactivated (Vero Cells)	Sinopharm (Wuhan), People’s Republic of China	2
	CoronaVac	Sinovac, Beijing, People’s Republic of China	53
	Turkovac	Health Institutes of Turkey, Istanbul, Turkey	1
	FAKHRAVAC (MIVAC)	Organization of Defensive Innovation and Research, Tehran, Iran	1
DNA	ZyCoV-D	Zydus Cadila, Ahmedabad, India	1

Notes: VVnr, non-replicating viral vector; PS, protein subunit; RNA, ribonucleic acid; DNA, deoxyribonucleic acid. Vaccines containing VVnr, RNA, and inactivated virus have become the most common. The main aspects of platforms for vaccine development are discussed below.

**Table 2:** Efficacy and safety of the most important COVID-19 vaccines.

Vaccine	Type	Dose Regimen	Prevention of Symptomatic Infection, % (95% CI)	Prevention of Severe Infection, % (95% CI)	Main Adverse Events
BNT162b2 [55,56]	mRNA	2 doses, 3-week interval	95.0 (90.3–97.6)	88.9 (20.1–99.7)	Pain, erythema, and swelling at the injection site Weakness, myalgia, chills
mRNA-1273 [57]	mRNA	2 doses, 4-week interval	93.2 (91.0–94.8)	98.2 (92.8–99.6)	Pain, erythema, and swelling at the injection site Axillary lymph node swelling and tenderness Fever, headache, weakness, myalgia, chills, nausea/vomiting
Ad26.COV2.S [58]	VV	Single dose	66.5 (55.5–75.1)	85.4 (54.2–96.9)	Pain, erythema, and swelling at the injection site Weakness, myalgia, chills
ChAdOx1 nCoV-19 [59]	VV	2 doses, 4-week interval	67.1 (52.3–77.3)	–	Pain, erythema, and swelling at the injection site Weakness, myalgia, chills
Sputnik V [21]	VV	2 doses, 3-week interval	91.1 (83.8–95.1)	100 (94.4–100)	Pain, erythema, and swelling at the injection site Weakness, myalgia, chills
BBIBP-CorV [60]	IV	2 doses, 4-week interval	78.1 (64.9–86.3)	–	Pain, erythema, and swelling at the injection site Fever, headache, cough
CoronaVac [61]	IV	2 doses, 4-week interval	83.5 (65.4–92.1)	–	Pain, erythema, and swelling at the injection site Weakness, myalgia, nausea, chills

Notes: VNA, virus neutralizing antibodies; CI, confidence interval; IV, inactivated virus; mRNA, messenger ribonucleic acid; VV, viral vector.
